# An instrument to assess quality of life in relation to nutrition: item generation, item reduction and initial validation

**DOI:** 10.1186/1477-7525-8-26

**Published:** 2010-03-11

**Authors:** Holger J Schünemann, Francesca Sperati, Maddalena Barba, Nancy Santesso, Camilla Melegari, Elie A Akl, Gordon Guyatt, Paola Muti

**Affiliations:** 1 Department of Medicine, University at Buffalo, State University of New York, Buffalo, New York, USA; 2 Department of Epidemiology, Italian National Cancer Institute Regina Elena, Rome, Italy; 3 Department of Clinical Epidemiology and Biostatistics, McMaster University, Hamilton, Ontario, Canada; 4 Barilla, SpA, Parma, Italy

## Abstract

**Background:**

It is arguable that modification of diet, given its potential for positive health outcomes, should be widely advocated and adopted. However, food intake, as a basic human need, and its modification may be accompanied by sensations of both pleasure and despondency and may consequently affect to quality of life (QoL). Thus, the feasibility and success of dietary changes will depend, at least partly, on whether potential negative influences on QoL can be avoided. This is of particular importance in the context of dietary intervention studies and in the development of new food products to improve health and well being. Instruments to measure the impact of nutrition on quality of life in the general population, however, are few and far between. Therefore, the aim of this project was to develop an instrument for measuring QoL related to nutrition in the general population.

**Methods and results:**

We recruited participants from the general population and followed standard methodology for quality of life instrument development (identification of population, item selection, n = 24; item reduction, n = 81; item presentation, n = 12; pretesting of questionnaire and initial validation, n = 2576; construct validation n = 128; and test-retest reliability n = 20). Of 187 initial items, 29 were selected for final presentation. Factor analysis revealed an instrument with 5 domains. The instrument demonstrated good cross-sectional divergent and convergent construct validity when correlated with scores of the 8 domains of the SF-36 (ranging from -0.078 to 0.562, 19 out of 40 tested correlations were statistically significant and 24 correlations were predicted correctly) and good test-retest reliability (intra-class correlation coefficients from 0.71 for symptoms to 0.90).

**Conclusions:**

We developed and validated an instrument with 29 items across 5 domains to assess quality of life related to nutrition and other aspects of food intake. The instrument demonstrated good face and construct validity as well as good reliability. Future work will focus on the evaluation of longitudinal construct validity and responsiveness.

## Background

The intake of food is a basic human need. This basic need is accompanied by sensations of both pleasure (e.g. related to taste, social interaction) and despondency (e.g. related to indigestion, gastrointestinal disturbances, weight gain). These sensations may affect quality of life (QoL) and may be influenced by different composition and nutrient content of food stuff.

Furthermore, certain nutrients and types of diets may be associated with other patient important outcomes such as longevity, mortality and morbidity. For instance, the Mediterranean diet and high fruit and vegetable intake may lead to a range of positive health outcomes (e.g. reduction in myocardial infarction, stroke and pulmonary disease) [1]. One could therefore argue that modification of diet, given its potential for positive health outcomes, should be widely advocated and adopted. However, the feasibility and success of dietary changes will depend, at least partly, on whether potential negative influences on QoL can be avoided. It is therefore important to assess how food intake and dietary changes relate to QoL. This is of particular importance in the context of dietary intervention studies and for the development of new food products to improve health and well being. In addition, QoL related to nutrition may potentially serve as a predictor of compliance with specific dietary interventions. Social context of nutrition, such as eating together, may impact on domains such as satisfaction and happiness [2].

Instruments to measure the impact of nutrition on quality of life in the general population, however, are few and far between [3]. For example, Hatton et al. found that a prepared diet improved nutritional health perceptions and affect and reduced hassles related to nutrition in patients with cardiovascular disease [4]. The authors used four tools that were modeled on disease specific quality of life and well-being instruments. While the instruments showed face validity and indicated that the measured outcomes improved, details of item generation and item reduction for these instruments were not described. Furthermore Hatton did not address quality of life related to social aspects of life, such as interaction with others during meals, in particular in societies that place high emphasis on diet and food intake.

Therefore, the aim of this project was to develop an instrument for measuring QoL related to nutrition and food intake in the general population.

## Methods

We followed standard methodology for quality of life instrument development based on the following six phases described in the framework by Guyatt and Kirshner [5]:

a. Identification of Population

b. Item Selection

c. Item Reduction

d. Item Presentation

e. Pretesting of Questionnaire

f. Validation of Questionnaire

**Figure 1:**
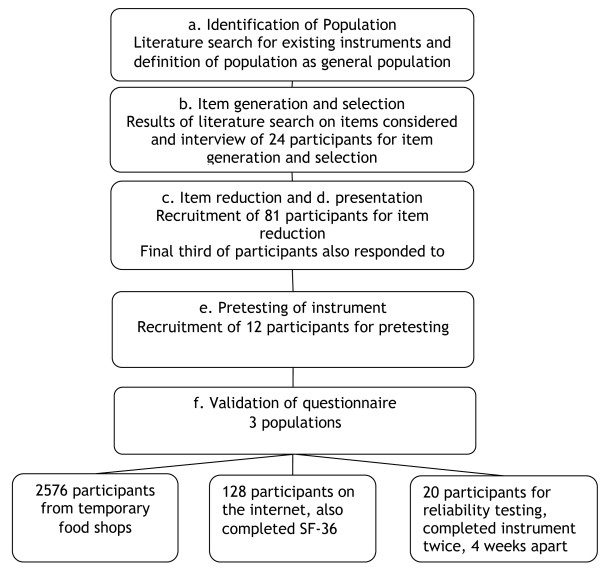
**Flow chart of phases in the study and recruited participants**.

We focused on discriminative properties in this study, but aimed to develop an instrument that eventually will be also useable for evaluative purposes. However, we did not investigate evaluative properties in this study. The flow of the study is described in figure 1.

### Search for existing instruments and identification of the population

We began our work by searching the literature for instruments measuring QoL related to food and food instruments related to QoL that could inform our work. We searched three databases (Medline, Health and Psychosocial Instruments (HAPI), CAB Abstracts) up to November 2007 and reviewed references from relevant articles (see search strategies in appendix 1). Of the 2083 citations resulting from the electronic search, there were two food related quality of life scales of particular interest that we reviewed prior to beginning the work on item generation [6,7]. Another citation described the use of questionnaires related to the impact of prepared diets on quality of life [4]. We also identified an abstract describing a nutrition QOL survey [8]. While providing potentially relevant items, these scales primarily focus on quality of life related to dietary therapy. Other instruments related to food provided potentially relevant themes such as food involvement, preparation, purchase, food diversity and social constraints related to food, but they focused on special populations. However, our focus was on the general population that might be exposed to general health messages regarding food intake and diet changes. Thus, we aimed to recruit a representative sample of the general population applying the following inclusion criteria: age greater than 18; no dietary restrictions (with the exception of vegetarian diet); able to read and speak Italian. We excluded participants with psychiatric, emotional or cognitive problems that could prevent reliable completion of the questionnaire; a diagnosis of a disease that is likely to influence completion of the questionnaire or selection of items; a major illness that substantially influences the patient's quality of life; distance of residence from recruitment centre of greater than 1 hour drive.

### Item generation and selection

This phase consisted of extraction of items from the reviewed literature, feedback from nutrition experts and semi-structured personal interviews (figure 1). Three investigators interviewed participants using a semi-structured questionnaire allocating up to 90 minutes to each interview. We recruited 24 participants in November and December 2007 through a consumer research agency in Rome, Italy. This consumer research agency holds a database of approximately 7000 individuals recruited since 1995 through public relation and publically available databases. Potential candidates were selected at random and invited by telephone or email to participate. Exclusion criteria for this phase of the study were work activity in the following professions: public relation, journalism, market research, marketing, food production or sales, psychology or sociology. Participants should not have participated in other interviews for at least 3 months prior to recruitment in this study. Participants received reimbursement for their travel expenses and provided informed consent. Table 1 shows the demographic characteristics of these participants.

**Table 1:** Demographic characteristics: item generation and selection phase (n = 24)

		mean	SD	Lowest - Highest
**Age**		30.5	7.53	19 - 44
**Weight**		70.7	16.1	46 - 112
**Height**		172.5	9.3	158 - 190
		**N**	**%**	
	
		24		
**Gender**				
			
	*M*	12	50.0	
	*F*	12	50.0	
**Level of education**				
			
	*Middle school*	14	58.3	
	*High school*	8	33.3	
	*Masters' level education*	1	4.2	
	*No Information*	1	4.2	
**Marital status**				
			
	*Married*	9	37.5	
	*Separated or divorced*	1	4.2	
	*Not married*	14	58.3	

The item generation phase generated 187 items that were partially overlapping.

### Item reduction and pretesting

We grouped the collected items into similar themes before recruiting 81 participants in four Italian cities (Rome, Naples, Parma and Milan) for item reduction and initial item presentation using the same agency as for the item generation phase in addition to participants who worked in food production and sales (figure 1). Table 2 shows the demographic characteristics of these participants. Participants used 7 point Likert-type scales to rate the relative importance (not important (1) to extremely important (7)), agreement with (complete disagreement (1) to complete agreement (7)), or frequency of occurrence (never (1) to always (7)) for each of the selected items. We also began testing comprehension of draft items in the final third of the 81 participants by obtaining feedback about readability and clarity of question.

**Table 2:** Demographic characteristics: item reduction and presentation phase (n = 81)

		mean	SD
**Age**		47.7	15.8
**Weight**		70.7	14.1
**Height**		167.4	13.7
		**N**	**%**
**Gender**			
	*M*	38	46.9
	*F*	43	53.1
**Level of education**			
	*Middle school*	21	26.2
	*High school*	47	58.8
	*University degree*	12	15.0
**Marital status**			
	*Married*	53	65.4
	*Separated or divorced*	4	4.9
	*Widow*	5	6.2
	*Never married*	19	23.5
**Type of employment**			
	*Stable work*	41	50.7
	*Term worker*	4	4.9
	*Unemployed*	2	2.5
	*Retired*	10	12.3
	*Homemaker*	13	16.0
	*Student*	11	13.6
**Smoker**			
	*Never smoker*	34	42.0
	*Current moker*	24	29.6
	*Former smoker*	23	28.4
**Diabetes mellitus type 2**			
	*No*	78	96.3
	*Yes*	3	3.7
**Cardiovascular disease**			
	*No*	76	93.8
	*Yes*	5	6.2
**Hypertension**			
	*No*	65	81.2
	*Yes*	15	18.8
**History of Cancer**			
	*No*	77	95.1
	*Yes*	4	4.9
**Following a special diet**			
	*No*	68	84.0
	*Yes*	13	16.0

We then conducted descriptive analysis, factor analysis, bivariate correlations and item-item correlations. After eliminating items of low importance, low agreement between participants about the importance or infrequent occurrence, we grouped the remaining 105 items in five domains (n = number of items per domain):

1) Sensations (n = 11)

2) Symptoms (n = 12)

3) Healthy lifestyle (n = 47)

4) Family function (n = 19)

5) Social and role function (n = 16)

An additional 12 items related to taste did not load on any of the domains. We then reduced the number of items in each domain by removing items with high inter-item correlations (r > 0.5) or items that covered similar aspects (e.g. separate items indicating that healthy food should prevent cancer, cardiovascular disease, hypertension and diabetes were grouped into preventing disease). We made the latter judgments through discussion and consensus of three investigators. The final list of items prior to item presentation included 31 items, two of which we considered possibly difficult to understand.

### Item presentation

We presented the 31 items resulting from item reduction to an additional 12 volunteers. All items required answers on a 7 point Likert-type scale and inquired about the past two weeks. As anticipated, participants did not easily understand two items and we dropped those items before pretesting and validation of the questionnaire. Participants showed ease of understanding and there were no obvious ceiling or floor effects for the remaining 29 items (the questionnaire, named Qualcibo, is shown in appendix 2). The instrument required less than approximately 12 minutes for completion.

### Validation of the questionnaire

We performed a number of validation exercises (figure 1). First, we recruited a sample of 2576 participants from the general population during introduction of a new food product in temporary shops in the cities of Milan and Rome. Potential participants entering the shops were approached by employees of the temporary shop to participate in the study. They were asked to sit down and complete a questionnaire, but we did not select information on participants who declined to participate. Participants were informed of the purpose of the study and completed the questionnaire on a computer touch screen.

Second, we recruited a sample of 128 participants on the internet through advertisement who completed the questionnaire online together with the Short Form 36 (SF-36) [9]. This recruitment was done in the context of an advertising campaign for the same new food product that was offered in the temporary shops. Advertisement was sent by email through a marketing agency that was responsible for marketing the new food product.

Third, 20 volunteers participated in a reliability study (figure 1). These participants were recruited as part of a study investigating biochemical markers of nutrient intake. They completed the questionnaire twice, approximately four weeks apart in the context of a clinic visit. No lifestyle changes were suggested to these individuals.

### Statistical analysis for validation and reliability study

We calculated Pearson correlation coefficients between the items selected for presentation of the initial validation set of 2576 participants. We based the allocation of the items of Qualcibo into domains on factor analysis (principal component analysis with varimax rotation) and face validity as judged by the investigators [10]. To investigate the internal consistency of Qualcibo domains, we calculated Cronbach alpha values [10]. We evaluated convergent and discriminant cross sectional construct validity by calculating Pearson's correlation coefficients between Qualcibo domains and the SF-36 domains in 128 participants. We considered correlations of less than 0.2 as very weak, from 0.2 to 0.35 as weak, from greater than 0.35 to 0.5 as moderate, and of more than 0.5 as strong. For interpretation of the data, we used *blinded *a priori ratings about the strength of the correlation between the 8 domains on the SF36 and the Qualcibo from four of the authors with significant experience in quality of life research. Finally, we evaluated the test-retest reliability of Qualcibo using repeated measurements in the sample of 20 individuals who completed the instrument twice. We calculated mean scores at the two administrations and compared these scores for all 29 questions using a paired t-test and calculated Pearson's correlation coefficient. We then calculated intra-class correlation coefficients by domain where the between-rater variance estimates at the two times of administration were in the numerator and the between-rater variance in addition to the within-rater variance of the two ratings in the denominator [11,12]. We used SPSS for Windows 14.0 and 17.0 for the statistical analyses (SPSS, Inc, Chicago, Ill).

### Ethics

The item generation and item reduction phase was approved by the ethics board of the Italian National Cancer Institute "Regina Elena" in Rome, Italy. Recruited participants signed an informed consent. For the latter part of the study, the requirement for informed consents was waived by the institutional review board and the reliability data were provided by one of the investigators (CM) as part of an ongoing study that had received ethics approval by the University of Parma.

## Results

### Development

Descriptive characteristics of the participants enrolled for the item generation and selection phase (n = 24) are shown in Table 1. 81 participants completed the extended item questionnaire of 187 items and their descriptive characteristics are shown in Table 2. We reduced this set of items to 29 through statistical analyses, discussion and item presentation in the item reduction phase.

### Pretesting and validation

Thirty-nine percent of the 2576 participants were men. The mean age of the recruited participants was 42.2 years, with a mean weight of 66.2 kg and height of 169.7 cm.

Table 3 shows the results of the factor analysis for the 29 items. The items loaded on five factors that were related to the initial clusters we identified: healthy lifestyle (n = 10 items), symptoms (n = 6), sensations (n = 6), social and role function (n = 4), taste (n = 3). Table 4 shows the internal consistency reliability for the entire set of questions and the single domains. Table 5 shows the mean scores for the five domains and table 6 the correlations among domains. The mean scores were above 4 (the mean of the score range) but the standard deviation was approximately 1.0 for all domains. We found that two items (becoming upset in relation to food intake and a feeling of happiness after a rich breakfast) were not loading uniquely on only one factor. Furthermore, one of these items (becoming upset in relation to food intake) showed a flat distribution indicating that this item may have been misunderstood by participants.

**Table 3:** Factor analysis of 29 items

	Factor				
**Item Nr. (Factor loading) and item name**	**1**	**2**	**3**	**4**	**5**

1. (2) avvertito pesantezza	.142	.788	.119	.016	-.104
2. (2) avvertito acidità/bruciore stomaco	.077	.653	.089	.004	.132
3. (1) evitato cibi pesanti/grassi/fritti	.729	.101	-.068	-.048	-.075
4. (2) avvertito sonnolenza	.259	.515	.011	.029	3.49E-005
5. (3) avvertito soddisfazione/sollievo morale	.005	.071	.724	.085	.074
6. (1) evitato grandi quantità di cibo	.663	.209	-.183	.040	-.020
7. (3) avvertito momento tranquillità	.131	.184	.649	.033	.082
8. (4) mangiato piatto nuovo	.032	-.043	.370	.410	-.159
9. (2) disturbi intestinali	.033	.697	.067	.014	.172
10. (1) controllato etichette/tipologia del cibo mangiato	.587	-.034	.228	-.040	.008
11. (4) occasione per riunirsi	.005	.025	.087	.844	.046
12. (1) fatto la spesa/partecipato alla preparazione del (pasto/rispettato stagionalità degli alimenti	.421	-.032	.177	.030	.259
13. (5) mangiato cibo con gusto che non piace	.143	.147	-.022	-.040	.597
14. (1) evitato di andare a dormire dopo mangiato/fatto una ((passeggiata	.345	.141	.014	.123	.111
15. (2) avvertito gonfiore	.109	.778	.153	.002	-.061
16. (1) seguito alimentazione che comprende tutti i gruppi ()alimentari	.720	.168	.112	.063	.062
17. (1) mangiato cibo sano	.597	.210	.171	.058	.236
18. (5) mangiato cibo con buon sapore	.104	.185	.266	.197	.631
19. (3) avvertito benessere personale/piacere	.070	.191	.711	.171	.289
20. (3) avvertito sensazione di recupero forze	.160	.160	.589	.098	-.006
21. (4) accordo sui gusti alimentari/cena ben cucinata ha migliorato la relazione con partner/famiglia	.157	.056	.360	.454	.127
22. (3) contento dopo abbondate colazione	.240	-.047	.318	.047	.126
23. (1) mangiato cibi che prevengono malattie	.581	.039	.288	.020	.007
24. (5) avvertito sazietà	-.051	-.159	.197	.054	.574
25. (1) controllato l'assunzione di cibi che fanno ingrassare	.729	.032	.037	-.029	-.010
26. (1) consumato prodotti di qualità	.690	.104	.187	.061	.042
27. (3) sentito odore di una petanza	.130	-.006	.491	.262	.340
28. (4) momento per stare in compagnia/parlare	.007	.053	.115	.827	.170
29. (2) avvertito cattivo umore in relazione a un pasto	.047	.478	.075	.110	.452

**Table 4:** Internal consistency reliability

	Cronbach alpha
Entire set of questions (n = 29)	0.86

Healthy lifestyle domain	0.83
Symptoms domain	0.77
Sensations domain	0.73
Social and role function domain	0.65
Taste domain	0.43

**Table 5:** Domain scores (n = 2576) of the five Qualcibo domains

	Minimum	Maximum	Mean	Std. Deviation
Healthy lifestyle	1	7	4.5	1.04
Symptoms	1	7	5.0	0.99
Sensations	1	7	4.5	0.95
Social and role function	1	7	4.2	0.96
Taste	1	7	5.3	0.92

**Table 6:** Domain-domain correlations (n = 2576)

	Healthy Lifestyle	Symptoms	Sensations	Social and role function
Healthy Lifestyle				
Symptoms	0.324(**)			
Sensations	0.358(**)	0.298(**)		
Social and role function	0.178(**)	0.141(**)	0.456(**)	
Taste	0.214(**)	0.225(**)	0.372(**)	0.232(**)

** all p values < 0.001

### Cross-sectional construct validity

The 128 participants (35.9% male) who participated in the internet survey and completed both the Qualcibo and the SF-36 had a mean age of 35.3 (SD 10.2) years, mean weight of 63.9 (SD 13.2) kg, and were 169.7 (SD 8.9, data missing on 14 individuals) cm tall. The correlations between the domains on the Qualcibo and the SF-36 ranged from -0.078 to 0.562 (table 7). Of the 40 tested correlations, 19 were statistically significant (p < 0.01 for 12 correlations and p < 0.05 for 7 correlations). The correlations in the Qualcibo symptoms domain with all of the SF36 domains were higher (primarily in the moderate to strong category) than the other Qualcibo domains. Most of the correlations were weak to very weak. However, except for the correlations with the symptoms domain for which we expected slightly lower correlations, the direction and magnitude of the associations were generally in line with the predictions by the authors with expertise in quality of life research. In fact, 24 correlations were predicted correctly, 15 were either higher or lower by one category and one lower by two categories (predicted as moderate correlation but resulted as very weak).

**Table 7:** Cross-sectional Construct Validity (n = 128)

	Healthy lifestyle	Symptoms	Sensations	Social and Role function	Taste
SF-36 Physical Function	.158	.366**	.123	.114	.235**
	.075	.000	.168	.199	.008

SF-36-Role Function	.053	.368**	.160	.025	.150
	.556	.000	.071	.777	.091

SF-36 Bodily Pain	-.022	.121	-.078	-.023	.108
	.803	.174	.384	.795	.226

SF-36 General Health	.124	.451**	.258**	.212*	.201*
	.162	.000	.003	.016	.023

SF-36 Vitality	.222*	.562**	.394**	.127	.269**
	.012	.000	.000	.154	.002

SF-36 Social Functioning	-.023	.504**	.171	.130	.199*
	.796	.000	.053	.144	.025

SF-36 Role-Emotional	.046	.458**	.138	.100	.195*
	.607	.000	.120	.260	.028

SF-36 Mental Health	.094	.538**	.361**	.209*	.192*
	.293	.000	.000	.018	.030

*p < 0.05; ** p < 0.01

### Reliability

The 20 participants who completed the Qualcibo twice had a mean age of 65.7 (SD 4.4) years, weighted 76.6 (SD 8.0) kg and were 166 (SD 7.8) cm tall. Despite performing 29 tests, there were no significant differences in the mean scores for any of the 29 questions between the two administrations. The correlation coefficients between the two administrations ranged from 0.03 to 0.82 and 16 correlations were above 0.5. The lowest correlation coefficient was largely driven by one respondent who reported a 7 on the first administration and a 1 on the second administration (item 15). The correlation coefficients by domain ranged from 0.55 to 0.84 (p < 0.05 for all domains). The intra-class correlation coefficients by domain were 0.84 for healthy lifestyle, 0.71 for symptoms, 0.90 sensations, 0.77 for social and role function, 0.73 for taste.

## Discussion

Applying standard methodology following an established framework, we created an instrument that evaluates quality of life related to nutrition [5]. The 29 items of the Qualcibo are simple to complete, show good face validity, and internal consistency reliability. Evaluation of construct validity generally indicated correlations with the SF-36 of expected magnitude and direction. Reliability of the instrument is also adequate. Our literature search indicated that validated instruments for the general population in this area are absent. We identified one abstract that described the development of a nutrition quality of life screening tool [8]. We therefore believe that this instrument may find application in nutrition surveys and clinical studies.

### Strength

We believe our study has several strengths. We started our work with a thorough review of the literature on existing items and generated a large list of candidate items. The extensive subsequent phases following standard methodology and using large sample sizes are another strength of this study.

### Limitations

This study has some limitations. First, the generalizability of the results need to be evaluated in an international context because this study was performed in only one Mediterranean country in only one language. Second, two items showed loading on more than one factor. For example, the item dealing with satisfaction and agreement on food taste (item 21) loaded on both the sensations and the social and role function domain. One possibility for this and similar instances is that the item actually does relate to more than one domain. Alternatively, despite the detailed efforts to ensure optimal phrasing of the item the intended question may not have been specific enough. This could result in differing understanding of the item across respondents. Third, the recruitment strategy might have favoured participants with an interest in nutrition. We believe that this could have led to higher than average scores on some of the domains. Finally, we only performed cross-sectional validation, but did not address longitudinal construct validity and responsiveness.

### Instrument properties

The instrument has 29 items with 5 domains: healthy lifestyle (n = 10 items), symptoms (n = 6 items), sensations (n = 6 items), social and role function (n = 4 items), and taste (n = 3 items). Mean scores were above 4 in the large validation set of 2576 participants who likely possessed above average interest in nutrition. Further work in other large representative populations is required to establish the mean score in the general population. However, we believe that the score distribution indicates that both deterioration and improvement will be detectable in most populations. Correlations with the SF-36 domains were very weak to strong. Although most of the correlations were weak, we expected these low correlations because our instrument focuses on domains that are only partially related to those of the SF-36 and more specific for food intake. Given that we made a priori predictions about the strength of the associations, the observed correlations indicated good construct validity. Finally, both internal consistency reliability and test-retest reliability indicate that this instrument has good psychometric properties.

### Context

In the context of recommendations about diet and clinical interventions to alter risk factors, the need for instruments to assess the impact of nutrition related lifestyle changes exists [3]. This instrument is one of the first to tackle the gap of validated tools to assess the relation between nutrition and quality of life. We found that sensations, symptoms healthy lifestyle, family function, social and role function are important in the context of nutrition. The impact on those domains should be considered in the prescription of dietary interventions to patients in both the clinical and the research settings. It will be important to explore whether potential small benefits in morbidity outcomes as a result of dietary interventions studies outweigh potential negative outcomes on quality of life and vice versa. Our instrument should allow this assessment. The instrument might also be able to predict the compliance of subjects with specific dietary interventions based on reported change in QoL with the introduction of those diets. The instrument will require additional work to ensure proper translation and cultural adaptation.

## Conclusions

We developed and validated an instrument to assess quality of life related to nutrition and other aspects of food intake. The instrument demonstrates promising validity and will be a suitable questionnaire for population based research on diet changes and the impact of nutrition on Qol. It can be used to determine whether dietary interventions negatively or positively influence individuals' perception of QoL related to nutrition. Further work will focus on the instruments longitudinal construct validity and responsiveness.

## Competing interests

The authors declare that they have no competing interests.

## Authors' contributions

HS had the original idea of developing this quality of life instrument, conceived the study, contributed to collecting all but the reliability data and analyzed data and wrote the first draft of this article. FS contributed to collecting all but the reliability data and analyzed data and reviewed the final draft of the article. MB contributed to collecting all but the reliability data and reviewed the final draft of the article. NS performed the literature search and reviewed the final draft of the article. CM approved the study protocol, supplied the data for the reliability study and reviewed the final draft of the article. EAA, GG, PM reviewed the study protocol, interpreted data and reviewed the final draft of the article. All authors read and approved the final draft of the manuscript.

## Appendix 1

### Search Strategies and Results

#### Health and Psychosocial Instruments 1985 to November 2007

1. (food$ or nutri$ or eat$ or feed$ or meal$ or diet$).m_titl.

2. (life or behavio$ or habit$ or practice$ or activit$ or attitud$ or belie$ or emotion$ or psych$).mp. [mp = title, acronym, descriptors, abstract]

3. (content$ or happ$ or satisf$ or quality or enjoy$ or pleas$).mp. [mp = title, acronym, descriptors, abstract]

4. 1 and (2 or 3)

1305 citations

#### Medline 1950 - November 2007

1. exp food/

2. exp nutrition therapy/

3. exp diet/

4. exp feeding behavior/

5. or/1-4

6. quality of life.tw.

7. quality of life/

8. ((content or contented$ or happy or happiness or happily or satisfy or satisfied or satisfaction or enjoy$ or pleas$) and life).tw.

9. or/6-8

10. psychometrics/

11. questionnaires/

12. (scale$ or questionnaire$).tw.

13. "Outcome Assessment (Health Care)"/

14. or/10-13

15. 5 and 9 and 14

683 citations

#### CAB Abstracts (1973 - November 2007)

1. (((DE "food" OR DE "food products" OR DE "foods" or DE "food beliefs" or DE "food intake" or DE "food intolerance (AGRICOLA)" or DE "food preferences" or DE "food preparation" or DE "food purchasing (AGRICOLA)" or DE "food quality" or DE "food research" or DE "food sciences" or DE "foods" OR DE "beverages" OR DE "carbohydrate-rich foods" OR DE "chewing gum" OR DE "confectionery" OR DE "convenience foods" OR DE "desserts" OR DE "dietetic foods" OR DE "ethnic foods (AGRICOLA)" OR DE "fast foods" OR DE "food pastes" OR DE "food supplements" OR DE "fried foods (AGRICOLA)" OR DE "functional foods" OR DE "garnishes (AGRICOLA)" OR DE "health foods" OR DE "infant foods" OR DE "kosher food (AGRICOLA)" OR DE "low acid foods (AGRICOLA)" OR DE "low calorie foods (AGRICOLA)" OR DE "low fat products" OR DE "natural foods (AGRICOLA)" OR DE "novel foods (AGRICOLA)" OR DE "organic foods" OR DE "pickled foods (AGRICOLA)" OR DE "precooked foods (AGRICOLA)" OR DE "protein foods" OR DE "salad dressings (AGRICOLA)" OR DE "salads (AGRICOLA)" OR DE sauces" OR DE "simulated foods" OR DE "soups" OR DE "spreads" OR DE "tropical foods (AGRICOLA)" OR DE "unconventional foods" OR DE "wild foods") and (DE "nutrition" or DE "nutrition knowledge" or DE "nutrition planning (AGRICOLA)" or DE "nutrition research" or DE "nutritional adequacy (AGRICOLA)" or DE "nutritional state")) or (DE nutrient intake (AGRICOLA)")) and (DE "diet" or DE "diet planning" or DE "dietetics")

2. ((DE "surveys" or DE "censuses" or DE "disease surveys" or DE "epidemiological surveys" or DE "household surveys" or DE "nutrition surveys" or DE "regional surveys (AGRICOLA)" or DE "data collection" or DE "research" or DE "sampling" or DE "surveillance" or DE "surveying") or (DE "measurement")) or (DE "dietary surveys")

3. AB tool OR instrument OR scale

4. 1 AND (2 OR 3)

95 citations

## Appendix 2

Questo questionario è concepito allo scopo di verificare come si è sentito/a nelle ultime 4 settimane. Per favore risponda a tutte le domande scegliendo una delle opzioni ed inserisca una X nella casella corrispondente alla risposta da Lei individuata. Non esistono risposte giuste o sbagliate. Nel caso in cui Lei fosse insicura/o riguardo a come rispondere ad una domanda, dia cortesemente la migliore risposta possibile. Le Sue risposte al presente questionario saranno trattate in modo confidenziale.

1. Indichi per favore quante volte nelle ultime 4 settimane in relazione all'assunzione di cibo Le è capitato di avvertire una sensazione di pesantezza.

|1| Sempre

|2| Quasi sempre

|3| Tante volte

|4| Qualche volta

|5| Poche volte

|6| Quasi mai

|7| Mai

2. Indichi per favore quante volte nelle ultime 4 settimane in relazione all'assunzione di cibo ha avvertito acidità o bruciore di stomaco.

|1| Sempre

|2| Quasi sempre

|3| Tante volte

|4| Qualche volta

|5| Poche volte

|6| Quasi mai

|7| Mai

3. Indichi per favore quanto spesso nelle ultime 4 settimane ha evitato cibi pesanti o cibi grassi e fritti.

|1| Non ho mai evitato cibi pesanti o cibi grassi e fritti

|2| Non ho quasi mai evitato cibi pesanti o cibi grassi e fritti

|3| Poche volte ho evitato cibi pesanti o cibi grassi e fritti

|4| Qualche volta ho evitato cibi pesanti o cibi grassi e fritti

|5| Tante volte ho evitato cibi pesanti o cibi grassi e fritti

|6| Ho quasi sempre evitato cibi pesanti o cibi grassi e fritti

|7| Ho sempre evitato cibi pesanti o cibi grassi e fritti

4. Indichi per favore quanto spesso nelle ultime 4 settimane in relazione all'assunzione di cibo Le è capitato di avvertire sonnolenza.

|1| Sempre

|2| Quasi sempre

|3| Tante volte

|4| Qualche volta

|5| Poche volte

|6| Quasi mai

|7| Mai

5. Indichi per favore quante volte nelle ultime 4 settimane in relazione all'assunzione di cibo Le è capitato di avvertire una sensazione di soddisfazione o sollievo morale.

|1| Mai

|2| Quasi mai

|3| Poche volte

|4| Qualche volta

|5| Tante volte

|6| Quasi sempre

|7| Sempre

6. Indichi per favore quante volte nelle ultime 4 settimane ha evitato grandi di consumare quantità di cibo.

|1| Non ho mai evitato grandi quantità di cibo

|2| Non ho quasi mai evitato grandi quantità di cibo

|3| Poche volte ho evitato grandi quantità di cibo

|4| Qualche volta ho evitato grandi quantità di cibo

|5| Tante volte ho evitato grandi quantità di cibo

|6| Ho quasi sempre evitato grandi quantità di cibo

|7| Ho sempre evitato grandi quantità di cibo

7. Indichi per favore quante volte nelle ultime 4 settimane in relazione all'assunzione di cibo Le è capitato di avvertire un momento di tranquillità.

|1| Mai

|2| Quasi mai

|3| Poche volte

|4| Qualche volta

|5| Tante volte

|6| Quasi sempre

|7| Sempre

8. Indichi per favore quanto spesso nelle ultime 4 settimane ha mangiato un piatto nuovo.

|1| Mai

|2| Quasi mai

|3| Poche volte

|4| Qualche volta

|5| Tante volte

|6| Quasi sempre

|7| Sempre

9. Indichi per favore quanto spesso nelle ultime 4 settimane ha consumato cibi che hanno creato disturbi intestinali.

|1| Sempre

|2| Quasi sempre

|3| Tante volte

|4| Qualche volta

|5| Poche volte

|6| Quasi mai

|7| Mai

10. Indichi per favore quanto spesso nelle ultime 4 settimane ha controllato le etichette dei cibi o controllato la tipologia del cibo che ha mangiato.

|1| Mai

|2| Quasi mai

|3| Poche volte

|4| Qualche volta

|5| Tante volte

|6| Quasi sempre

|7| Sempre

11. Indichi per favore quante volte nelle ultime 4 settimane l'assunzione di cibo è stata per Lei un occasione per riunirsi.

|1| Mai

|2| Quasi mai

|3| Poche volte

|4| Qualche volta

|5| Tante volte

|6| Quasi sempre

|7| Sempre

12. Indichi per favore quante volte nelle ultime 4 settimane ha fatto personalmente la spesa, preparato o partecipato alla preparazione di un pasto caldo per la sua famiglia o rispettato la stagionalità degli alimenti nel preparare un pasto.

|1| Mai

|2| Quasi mai

|3| Poche volte

|4| Qualche volta

|5| Tante volte

|6| Quasi sempre

|7| Sempre

13. Indichi per favore quanto spesso nelle ultime 4 settimane ha mangiato un cibo con un gusto che non Le piace.

|1| Non ho mai mangiato cibo di mio gusto

|2| Non ho quasi mai mangiato cibo di mio gusto

|3| Poche volte ho mangiato cibo di mio gusto

|4| Qualche volta ho mangiato cibo di mio gusto

|5| Tante volte ho mangiato cibo di mio gusto

|6| Ho quasi sempre mangiato cibo di mio gusto

|7| Ho sempre mangiato cibo di mio gusto

14. Indichi per favore quanto spesso nelle ultime 4 settimane ha evitato di andare subito a dormire dopo aver mangiato o ha fatto una passeggiata dopo aver mangiato troppo.

|1| Mai

|2| Quasi mai

|3| Poche volte

|4| Qualche volta

|5| Tante volte

|6| Quasi sempre

|7| Sempre

15. Indichi per favore quante volte nelle ultime 4 settimane in relazione all'assunzione di cibo Le è capitato di avvertire una sensazione di gonfiore.

|1| Sempre

|2| Quasi sempre

|3| Tante volte

|4| Qualche volta

|5| Poche volte

|6| Quasi mai

|7| Mai

16. Indichi per favore quanto spesso nelle ultime 4 settimane ha mangiato leggero o seguito un'alimentazione che comprenda tutti i gruppi alimentari.

|1| Mai

|2| Quasi mai

|3| Poche volte

|4| Qualche volta

|5| Tante volte

|6| Quasi sempre

|7| Sempre

17. Indichi, per favore, quante volte nelle ultime 4 settimane nella sua opinione ha consumato cibo sano.

|1| Mai

|2| Quasi mai

|3| Poche volte

|4| Qualche volta

|5| Tante volte

|6| Quasi sempre

|7| Sempre

18. Indichi, per favore, quante volte nelle ultime 4 settimane ha consumato cibo con un buon sapore. Scelga una delle seguenti opzioni:

|1| Mai

|2| Quasi mai

|3| Poche volte

|4| Qualche volta

|5| Tante volte

|6| Quasi sempre

|7| Sempre

19. Quante volte nelle ultime 4 settimane in relazione all'assunzione di cibo Le è capitato di avvertire una sensazione di benessere personale o di piacere?

|1| Mai

|2| Quasi mai

|3| Poche volte

|4| Qualche volta

|5| Tante volte

|6| Quasi sempre

|7| Sempre

20. Indichi per favore quante volte nelle ultime 4 settimane in relazione all'assunzione di cibo Lei ha avvertito nella giornata una sensazione di recupero delle forze?

|1| Mai

|2| Quasi mai

|3| Poche volte

|4| Qualche volta

|5| Tante volte

|6| Quasi sempre

|7| Sempre

21. Indichi, per favore, quanto spesso nelle ultime 4 settimane l'accordo sui gusti alimentari o una cena ben cucinata ha migliorato la relazione con il partner o la famiglia.

|1| Mai

|2| Quasi mai

|3| Poche volte

|4| Qualche volta

|5| Tante volte

|6| Quasi sempre

|7| Sempre

22. Indichi, per favore, quanto spesso nelle ultime 4 settimane si è sentito contento/a di aver fatto un' abbondante colazione.

|1| Mai

|2| Quasi mai

|3| Poche volte

|4| Qualche volta

|5| Tante volte

|6| Quasi sempre

|7| Sempre

23. Indichi per favore quanto spesso nelle ultime 4 settimane nella sua opinione ha consumato cibi che prevengono malattie.

|1| Mai

|2| Quasi mai

|3| Poche volte

|4| Qualche volta

|5| Tante volte

|6| Quasi sempre

|7| Sempre

24. Indichi per favore quante volte nelle ultime 4 settimane in relazione all'assunzione di cibo Le è capitato di avvertire una sensazione di sazietà.

|1| Mai

|2| Quasi mai

|3| Poche volte

|4| Qualche volta

|5| Tante volte

|6| Quasi sempre

|7| Sempre

25. Indichi, per favore, quanto volte nelle ultime 4 settimane ha potuto controllare l'assunzione di cibi che fanno ingrassare.

|1| Mai

|2| Quasi mai

|3| Poche volte

|4| Qualche volta

|5| Tante volte

|6| Quasi sempre

|7| Sempre

26. Indichi, per favore, quanto spesso nelle ultime 4 settimane ha consumato prodotti di qualità che La mantengono in forma.

|1| Mai

|2| Quasi mai

|3| Poche volte

|4| Qualche volta

|5| Tante volte

|6| Quasi sempre

|7| Sempre

27. Indichi per favore quanto spesso nelle ultime 4 settimane Lei ha avvertito piacere quando ha sentito o annusato l'odore di una pietanza.

|1| Mai

|2| Quasi mai

|3| Poche volte

|4| Qualche volta

|5| Tante volte

|6| Quasi sempre

|7| Sempre

28. Indichi per favore quante volte nelle ultime 4 settimane l'assunzione di cibo è stata per Lei un momento per stare in compagnia o parlare.

|1| Mai

|2| Quasi mai

|3| Poche volte

|4| Qualche volta

|5| Tante volte

|6| Quasi sempre

|7| Sempre

29. Indichi, per favore, quanto spesso nelle ultime 4 settimane Le è capitato di avvertire un cattivo umore in relazione a un pasto.

|1| Sempre

|2| Quasi sempre

|3| Tante volte

|4| Qualche volta

|5| Poche volte

|6| Quasi mai

|7| Mai
